# Genetic diversity and antiretroviral resistance-associated mutation profile of treated and naive HIV-1 infected patients from the Northwest and Southwest regions of Cameroon

**DOI:** 10.1371/journal.pone.0225575

**Published:** 2019-11-21

**Authors:** Henry Dilonga Meriki, Kukwah Anthony Tufon, Damian Nota Anong, Pascal Nji Atanga, Irene Ane Anyangwe, Fidelis Cho-Ngwa, Theresa Nkuo-Akenji

**Affiliations:** 1 Department of Microbiology and Parasitology, University of Buea, Buea, SW Region, Cameroon; 2 BioCollections Worldwide Inc., Regional Office, Buea, SW Region, Cameroon; 3 Department of Biochemistry and Molecular Biology, University of Buea, Buea, SW Region, Cameroon; 4 Cameroon Baptist Convention Health Service, Mutengene, South West Region, Cameroon; 5 Laboratory Department, Buea Regional Hospital, Buea, SW Region, Cameroon; University of Cincinnati College of Medicine, UNITED STATES

## Abstract

**Background:**

Antiretroviral therapy (ART) has improved the survival of HIV infected persons. However, rapid scale-up of ART and the high HIV-1 genetic variability, has greatly influenced the emergence of drug-resistant strains. This constitutes a potential threat to achieving the UNAIDS’ 90-90-90 goals by 2020. We investigated the prevalent HIV-1 genotypes, drug resistance-associated mutations and assessed some predictors of the occurrence of these mutations.

**Methods:**

This was a hospital-based cross-sectional study conducted between October 2010 and June 2012. Participants were consecutively enrolled from selected HIV treatment centers of the Southwest and Northwest regions of Cameroon. Viral load was determined with the automated Abbott Real-time HIV-1 m2000rt System. HIV genotyping and antiretroviral resistance mutations analysis were performed using Bayer’s HIV-1 TRUGENE^™^ Genotyping Kit and OpenGene DNA Sequencing system. The drug resistance mutation was interpreted with the Stanford HIV database. Epidemiological data were obtained using pre-tested semi-structured questionnaires.

**Results:**

Of the 387 participants, 239 were successfully genotyped. The median age of these participants was 33 years (interquartile range, IQR: 28–40 years), and a majority (65.7%) were female. A total of 29.3% of the participants were receiving ART. The median duration of ART was 10.5 months (IQR: 4–17.25 months). The median CD4 count and log_10_ viral load of study participants were 353.5 cells/ml (IQR:145–471) and 4.89 copies/ml (IQR: 3.91–5.55) respectively. CRF02 (A/G) (69%) was the most prevalent subtype followed by G (8.2%) and F (6.7%). Overall, resistance mutations were present in 37.1% of ART-experienced and 10.7% of ART-naive patients. Nucleoside reverse transcriptase inhibitors (NRTI) mutations occurred in 30% of ART-experienced and 2.4% of ART-naïve patients, while non-nucleoside reverse transcriptase inhibitors (NNRTI) mutations occurred in 34.2% of ART-experienced and 10.1% of -naïve patients. M184V (8.4%, 20/239) and K103N (5.4%, 13/239) were the most prevalent mutations. Major protease inhibitor mutations occurred in 3 (1.3%) out of the 239 sequences. The duration of ART independently predicted the occurrence of resistance mutation among ART-experienced patients.

**Conclusion:**

The high resistance to NNRTIs, which are the main support to the backbone (NRTIs) first-line antiretroviral regimen in Cameroon, has prompted the need to rollout an integrase strand transfer inhibitor regimen (containing Dolutegravir) with a higher genetic barrier to resistance as the preferred first line regimen.

## Introduction

Although preventable, HIV infection continues to be a major global public health concern. At the end of 2017, approximately 36.9 million people were living with HIV, with 1.8 million new infections [1]. The WHO African Region is the most affected region, accounting for 70% of all people living with HIV (PLHIV) [1]. HIV genetic diversity is one of its most significant features that may directly influence the global distribution, vaccine design, therapy success rate, disease progression, transmissibility [2] and the emergence of drug-resistant strains [3]. The high rate of error-prone viral replication accounts for this genetic diversity [4].

The less pathogenic HIV-2 can be found mainly in West Africa while HIV-1 is widely distributed across the world [5] and is responsible for the AIDS pandemic. HIV-1 strains have undergone extensive genetic alterations [6] and this has given rise to numerous genetically diversified strains, subtypes and sub-sub types [7]. Most sequences in group M viruses fall within a limited number of discrete clades and this allows the classification of HIV-1 M strains into 9 subtypes: A, B, C, D, F, G, H, J and K [8]. Some subtypes exhibit further distinct sequence clusters giving rise to sub-subtypes [9,10]. In addition to this, it became evident from phylogenetic analysis that some isolates combined with different subtypes in different regions of their genomes to give rise to mosaic HIV-1 genomes referred to as circulating recombinant forms (CRF) [8]. At least 51 CRFs have been identified [10] with a significant proportion presently found in Africa alongside all the other groups and subtypes [7]. Subtype C has continued to predominate in the Southern parts of Africa (South Africa, Zambia and Zimbabwe) [9] while subtype B is seemingly the most common in North Africa (Morocco, Egypt, Algeria) [11–13]. West and Central African countries have a wide distribution of HIV-1 M subtypes and Cameroon probably harbours the highest number of HIV-1 subtypes [10]. Furthermore, HIV-1 group N [14] as well as the new putative HIV-1 group P [15,16], have been reported only in Cameroon. In all the regions of Cameroon, genetic diversity seems to be high in both rural and urban areas despite regional differences in strain prevalence [17–20].

Additionally, the high genetic variability of HIV-1 may favour the development of antiretroviral drug resistance [3]. Although combined antiretroviral therapy seems to be effective against all HIV-1 subtypes, emerging evidence suggests global differences in HIV-1 subtypes may impact drug resistance and this may be relevant to ART strategies in specific settings [21]. For example, studies have shown, subtype C may acquire the tenofovir-related mutation K65R more rapidly when compared to subtype B [22,23], while mutations associated with resistance to rilpivirine are rare in infected patients with HIV-1 subtypes CRF01-A/E failing a first-line NNRTI-containing regimen [24]. HIV-1 genetic recombination is also a potential mechanism that favours the development of drug resistance although the impact on the clinical outcomes of ART is unclear [25]. HIV drug resistance is therefore, a real challenge for many countries including Cameroon in meeting the UNAIDS’ 90-90-90 goals by 2020 [26].

ART is life-long and consequently, adherence is key to the success of the ART outcome. Sub-optimal adherence to ART is associated with increased morbidity and mortality resulting from failure to achieve sustained viral suppression, drug resistance and potential transmission of the drug-resistant virus [27]. Although African HIV/AIDS patients have similar or higher adherence levels compared to those of developed countries [28], they are faced with many challenges related to their socioeconomic status, medication, and healthcare systems that affect their adherence to ART. This study, therefore, investigated the HIV-1 genetic diversity and prevalent resistance-associated mutations as well as some predictors of the occurrence of these mutations.

## Materials and methods

### Study population and sample collection

This was a cross-sectional, hospital-based study conducted between October 2010 and June 2012. The target population was HIV infected participants who were consecutively enrolled from the HIV treatment centers of Limbe Regional Hospital, Buea Regional Hospital, Tiko Central Clinic, and Kumba District Hospital, in the Southwest region. In the Northwest region, participants were enrolled from St. Theresa Catholic Medical Centre (STCMC)-Mambu-Bafut and Bamenda Regional Hospital. A pre-tested semi-structured questionnaire was used to collect data on demographic, socioeconomic, and behavioural characteristics from participants’ medical records and through face-to-face interviews.

About five milliliters of blood was collected in ethylene diamine tetraacetate (EDTA) tubes from every participant and used for laboratory analyses. CD4+T-cell counts were performed with BD Biosciences FACSCount^™^, (New Jersey, USA) following the manufacturers’ instructions. Samples were processed for plasma by centrifugation at 1100g for 20 minutes. Aliquots of plasma were stored at -20°C and shipped (in dry ice) to BioCollections Worldwide Inc., Miami, Florida, USA for HIV viral load and antiretroviral drug-resistant mutation analysis.

### Viral load analysis and genotyping

Plasma viral load was determined with the automated Abbott RealTime HIV-1 m2000rt System following the manufacturer’s instructions. Samples with a viral load above the lower limit of detection (40 copies/ml) were further genotyped. HIV genotyping and ARV resistance mutations analyses were performed using Bayer’s HIV-1 TRUGENE^™^ Genotyping Kit and OpenGene DNA Sequencing System (Siemens Healthcare Diagnostics Inc. Deerfield, Illinois, USA) according to the manufacturer’s instructions. The Bayer’s HIV-1 TRUGENE^TM^ Genotyping Kit amplifies and sequence codons 4 to 99 of the protease gene and codons 38 to 247 of the reverse transcriptase gene of the pol region of the HIV-1 genome. The sequences were confirmed on the Stanford University HIV Drug resistance database, Geno2pheno resistance database (Max Plank Institute) and HIV sequence database (Los Alamos, National Laboratory). The HIV-1 major drug resistance mutations obtained were interpreted on the Stanford HIV database program available on the University of Stanford HIV Drug resistance website. (http://sierra2.stanford.edu/sierra/servlet/JSierra

### Phylogenetic analysis

Multiple sequence alignments were obtained by codon-alignment with the CLUSTAL omega algorithm. The phylogenetic analysis was inferred using the Neighbor-Joining method conducted on MEGA X [29]. Reference sequences included in the phylogenetic analysis were downloaded following a blast from National Center for Biotechnology Information web site. https://www.ncbi.nlm.nih.gov/.

### Data analysis

Data analysis was performed with IBM SPSS 23.0 (Statistical Package for Social Sciences, Chicago, Illinois). Data were presented as counts, median and interquartile range (IQR). Univariate analysis was performed with the Chi-square test. Odds ratios (ORs) and nominal 95% confidence intervals (CIs) were presented. A multivariable logistic regression model was used to estimate the association between baseline demographic, clinical, socioeconomic and behavioural determinants and the occurrence of drug resistance-associated mutations. Only significant variables were included in the final model. A two-sided p-value < 0.05 was considered significant.

### Ethical statement

Administrative authorisations were obtained from the Directors of the health facilities used in this study. The ethical approval for the study was provided by the Cameroon National Ethics Committee (N^o^: 21/CNE/SE/2010). Consent was both verbal and written and each participant signed an informed consent form. Viral load and drug resistance results were made available to the healthcare workers dispensing ARVs. However, we are not certain the treating clinicians used these results in the management of patients at the time this study was conducted.

## Results

### Study population characteristics

The study enrolled 239 HIV-positive participants with a viral load above 40 copies/ml and whose samples were successfully amplified from a total of 387. The age of the participants ranged from 19–62 years and a median age of 33 years (IQR: 28–40 years). The majority of the participants were females (65.7%) and between the ages of 30–45 years (52.3%). A total of 29.3% of the participants were receiving antiretroviral therapy (ART-experienced). The median duration of ART was 10.5 months (IQR: 4–17.25 months) with most of the participants on nevirapine-based regimen (62.9%). Of the 169 ART-naïve patients, 54.4% were newly diagnosed. The median CD4 counts and log_10_ viral load of study participants were 353.5 cells/μl (IQR: 145–471) and 4.89 copies/ml (IQR: 3.91–5.55) respectively. The median CD4 counts of ART-experienced and ART-naïve patients were 240 cells/μl (IQR: 145–410) and 405 cells/μl (IQR: 146–546) respectively. While the log_10_ viral load for ART-experienced and ART-naïve patients were 3.53 copies/ml (IQR: 2.98–4.07) and 5.20 copies/ml (IQR: 4.67–5.72) respectively (Table 1).

**Table 1:** Baseline characteristics of study participants (n = 239).

Characteristics	Categories	Frequency (%)
**Gender**	Male	82 (34.3)
	Female	157 (65.7)
**Median age (years)**	33 (Interquartile range, IQR: 28–40)	
	ART-experienced:	31 (IQR: 29–40)
	ART-naïve:	33 (IQR: 28–41)
**Age groups (years)**	19–29	75 (31.4)
	30–40	105 (43.9)
	> 40	59 (24.7)
**ART status**	Treated	70 (29.3)
	Naive	169 (70.7)
**Regimen type**	**Nevirapine-based (n = 44)**	
	d4T/3TC/NVP	36 (51.4)
	AZT/3TC/NVP	8 (11.4)
	**Efavirenz-based (n = 26)**	
	AZT/3TC/EFV	17 (24.3)
	d4T/3TC/EFV	9 (12.9)
**Median duration of ART:**	10.5 months (IQR: 4.0–17.3 months)	
**Median CD4 (cells/μl):**	353.5 (IQR:145–471)	
	ART-Experienced	240 (IQR: 145–410)
	ART-Naïve	405 (IQR: 146–546)
**Median Log**_**10**_ **viral load (copies/ml):**	4.89 (IQR: 3.91–5.55)	
	ART-Experienced	3.53 (IQR: 2.98–4.07)
	Art-Naïve	5.20 (IQR: 4.67–5.72)

AZT-Zidovudine, 3TC-Lamivudine, NVP-Nevirapine, 4dT-Stavudine, EFV-Efavirenz

### Genetic diversity of HIV-1 subtypes

**Fig 1:**
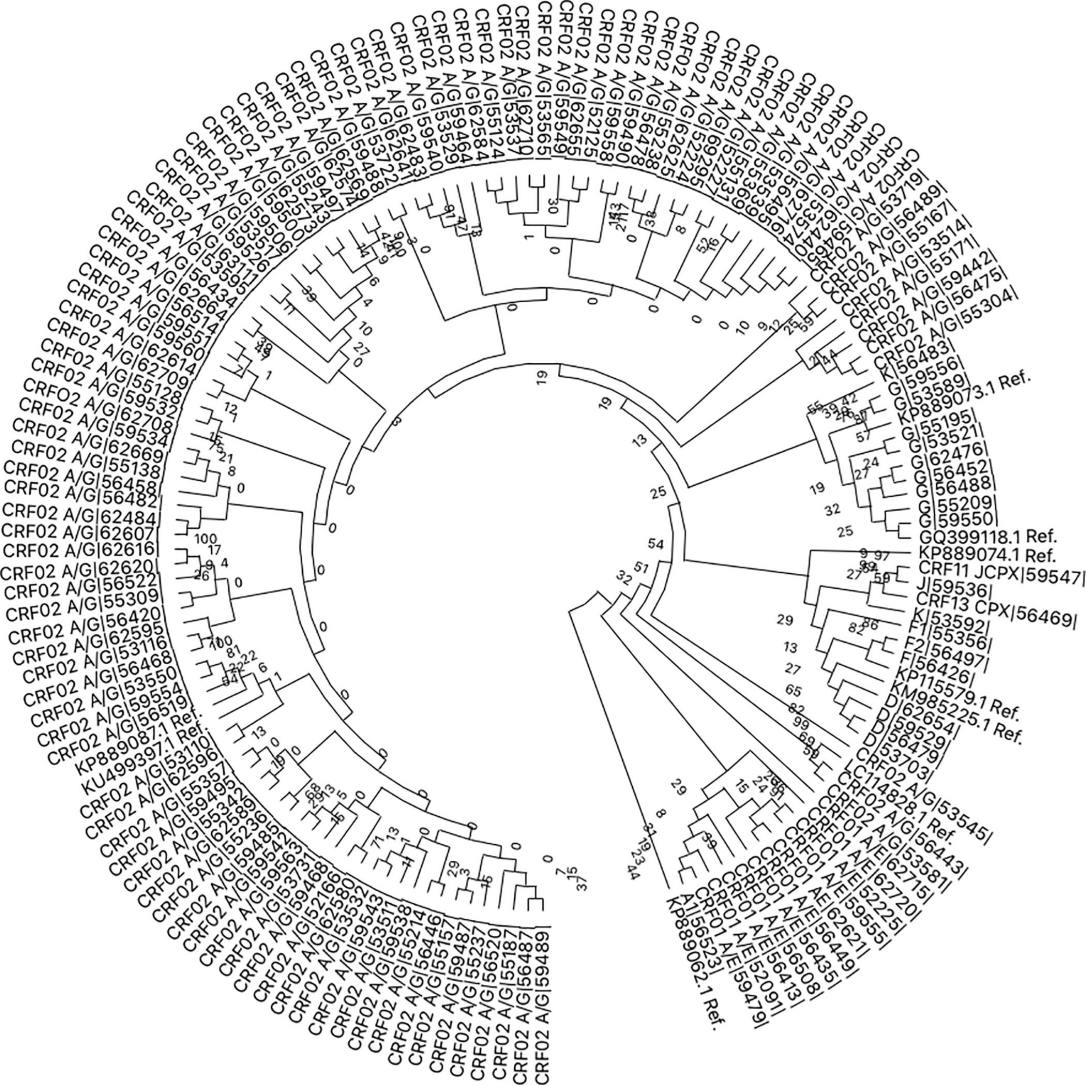
Phylogenetic analysis of the HIV-1 pol region based on neighbour-joining methods was constructed with MEGA X software. The codes of the reference sequences have the acronym “Ref.” at the end. The HIV-1 subtype for each sequence is written at the beginning of the code followed by the sample number in the bitwise sign. (For example D|56479|. Where D is the subtype and |56479| is the sample code).

The subtype CRF_02 (A/G) (68.6%, 165/239) was the most prevalent followed by G (8.2%, 20/239), F (6.7%, 16/239), A (4.6%, 11/239) and D (3.8%, 9/239) (Fig 1).

### Prevalence of drug resistance mutations

**Fig 2:**
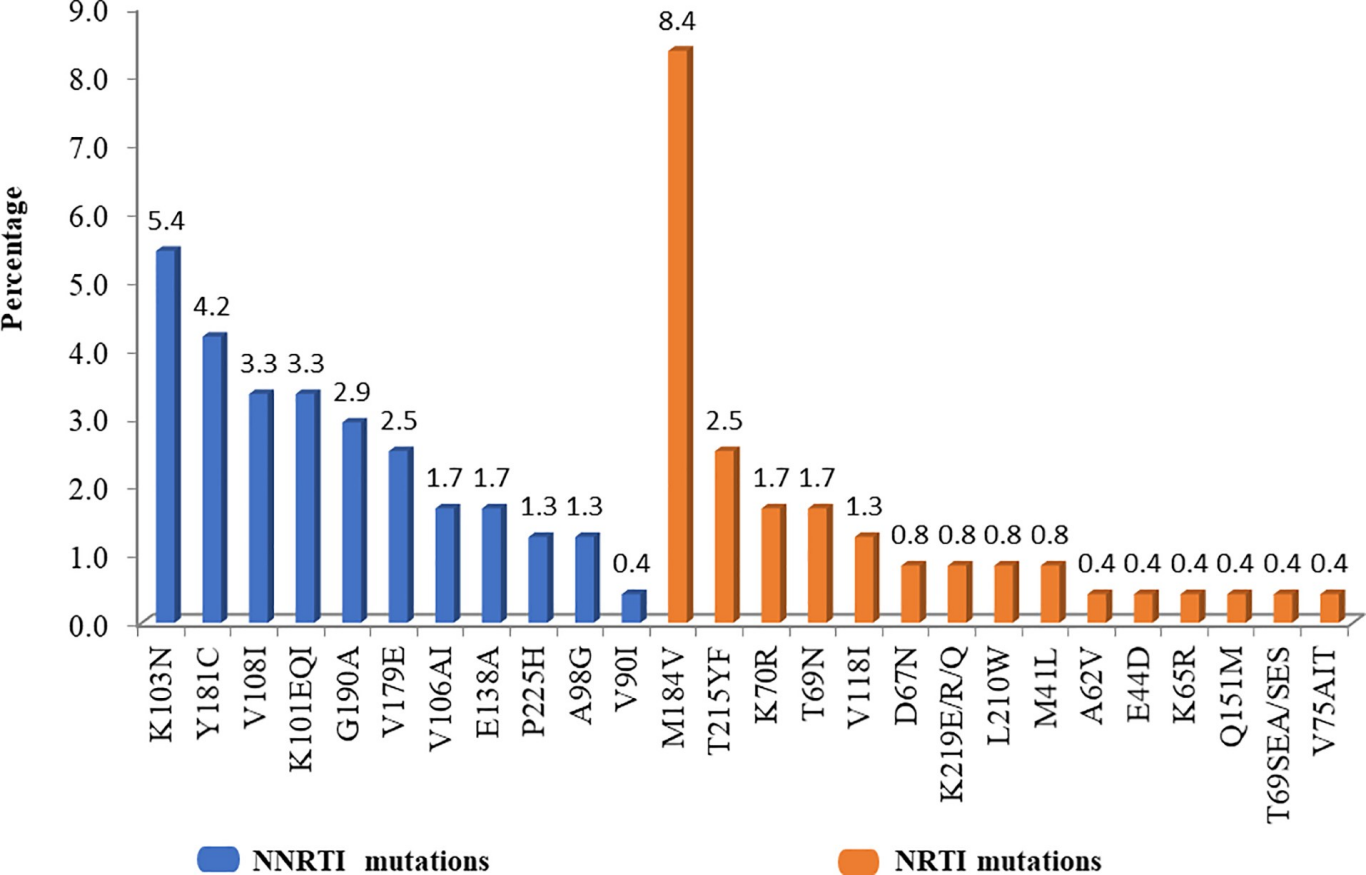
Prevalence of nucleoside reverse transcriptase inhibitor (NRTI) and non-nucleoside reverse transcriptase inhibitor (NNRTI) mutations.

Reverse transcriptase resistance mutations (RTRMs) were present in 44 (18.4%, 95% confidence interval (CI): 13.5% - 23.3%) of the 239 sequences analysed. Of the 70 participants in the ART-experienced group, the prevalence of resistance mutations was significantly higher (p = 0.017) in patients on ARTs for ≥ 6 months (47.7%, 21/44) compared to those < 6 months (19.2%, 5/26). Whereas, 18 (10.7%, 95% CI: 6.0%– 15.4%) of the 169 ART-naive patients harboured these RTRMs. Overall, the prevalence of nucleotide reverse transcriptase inhibitors (NRTI) and non-nucleoside reverse transcriptase inhibitors (NNRTI) resistance mutations were 10.5% (25/239) and 17.2% (41/239) respectively. Fifty per cent (22/44) of the sequences with RTRMs had mutations to both NRTI and NNRTI. Nineteen (43.2%, 19/44) had resistance mutations to NNRTI only, and 3 (6.8%, 3/44) had mutations to NRTI only. NRTI-associated mutations were present in 30% (21/70) and 2.4% (4/169) of ART-experienced and ART-naïve patients respectively. On the other hand, the prevalence of NNRTI-associated mutations in ART-experienced and ART-naïve patients was 34.2% (24/70) and 10.1% (17/169) respectively. M184V (8.4%, 20/239) and K103N (5.4%, 13/239) were the most frequent NRTI- and NNRTI-associated mutations respectively. Eleven (25%, 11/44) of the RTRM were thymidine analogue mutations (T215FY [2.5%, 6/239], K70R [1.7%, 4/239] and 0.8% (2/239) each of D67N, L210W, M41L, and K219Q/E (Fig 2).

Major protease inhibitor mutations were present in 3 (1.3%, 95% CI: 0.3–3.6%) of the sequences occurring as I54T-V82A, M46I and M46LV. All three participants were not treated with protease inhibitors antiretroviral. Minor PI mutations were present in 27.6% (66/239) of the sequences with L10I/V being the most prevalent (22.2%) mutation followed by V11I (4.6%) and E35G (2.5%).

### Drug resistance mutational patterns based on Stanford HIV database interpretation

Twenty of the 25 sequences with NRTI mutations conferred high-level resistance (HLR) to at least a drug in the class, with 10 major (presence of at least one HLR) resistance patterns. The most frequent pattern was M184V only (36%, 9/25) resulting in HLR to lamivudine/emtricitabine, low-level resistance (LLR) to abacavir and potential low-level resistance (PLR) to didanosine. The mutational pattern, D67N-M41L-M184V-L210W-T215F/Y was the most resistant conferring HLR to all drugs in the class, except tenofovir that showed intermediate resistance (IR) (Table 2).

**Table 2:** Resistance patterns of the 25 sequences with nucleoside reverse transcriptase inhibitor (NRTI) mutations analyzed on the Stanford HIV database.

Mutational patterns		Drug resistance profile						
	No (%)	^&^3TC	^¢^FTC	^š^ABC	^¶^ AZT	^¥^d4T	^μ^ddL	^+^TDF
**ART-Experienced (n = 21)**								
D67N-M41L-M184V-L210W-T215F/Y	1 (4.0)	*HLR	HLR	HLR	HLR	HLR	HLR	IR
D67N-K70R-M184V-T215Y-K219E/Q	1 (4.0)	HLR	HLR	IR	HLR	HLR	IR	^ø^LLR
M184V-L210W-T215Y	1 (4.0)	HLR	HLR	IR	HLR	HLR	IR	LLR
A62V-K65R-V75A/I/T-M184V	1 (4.0)	HLR	HLR	HLR	S	HLR	HLR	^†^IR
Q151M-M184V	1 (4.0)	HLR	HLR	HLR	IR	IR	HLR	^#^S
M184V-T215F	1 (4.0)	HLR	HLR	IR	IR	IR	LLR	S
K70R-M184V	1 (4.0)	HLR	HLR	LLR	LLR	S	^‡^PLR	S
T69SEA/SES-M184V-T215Y	1 (4.0)	HLR	HLR	IR	IR	IR	LLR	S
K70R-M184V-T69N	2 (8.0)	HLR	HLR	LLR	LLR	PLR	LLR	S
M184V	8 (32.0)	HLR	HLR	LLR	S	S	PLR	S
V118I, K219ER	1 (4.0)	S	S	PLR	LLR	LLR	PLR	S
M41L	1 (4.0)	S	S	S	LLR	LLR	S	S
T69N	1 (4.0)	S	S	S	S	S	PLR	S
**ART-Naïve (n = 4)**								
M184V-T215F	1 (4.0)	HLR	HLR	IR	IR	IR	LLR	S
M184V	1 (4.0)	HLR	HLR	LLR	S	S	PLR	S
T69N	1 (4.0)	S	S	S	S	S	PLR	S
E44D	1 (4.0)	S	S	S	S	S	S	S

^&^3TC: Lamivudine

^š^ABC: Abacavir

^¶^ AZT: Zidovudine

^¥^d4T: Stavudine

^μ^ddl: Didanosine

^¢^FTC: Emtricitabine

^+^TDF: Tenofovir

*HLR: high-level resistance

^ø^LLR: Low-level resistance

^†^IR: intermediate resistance

^‡^PLR: Potential Low-level resistance

^#^S: susceptible.

Mutations conferring high-level resistance (HLR) to at least a drug in the NNRTI class were present in 63.4% (26/41) of the sequences. The most common patterns among ART-experience patients were K103N only or in combination with P225H or V106A (9.8%, 4/41), Y181C only or in combination with V108I (9.0%, 4/41) and V179E (4.9%, 2/41). The most resistance NNRTI-associated pattern was G190A-Y181C which showed HLR to all the drugs in the class and this was from an ART-naive patient (Table 3).

**Table 3:** Resistance patterns with non-nucleoside reverse transcriptase inhibitor mutations (NNRTI) analyzed on the Stanford database (n = 41).

Mutational pattern		Drug resistance profile			
	No (%)	EFV^&^	NVP^¶^	ETR^š^	RPV^μ^
**ART-Experienced (n = 24)**					
A98G-K103N-Y181C	1 (2.4)	HLR*	HLR	IR^†^	IR
K103N-Y181C	1 (2.4)	HLR	HLR	IR	IR
A90G-G190A-K103N-V108I	1 (2.4)	HLR	HLR	LLR^ø^	LLR
K101E-V108I-Y181C	1 (2.4)	HLR	HLR	LLR	LLR
G190A-K101E	1 (2.4)	HLR	HLR	LLR	LLR
K103N-V179E	1 (2.4)	HLR	HLR	PLR^‡^	PLR
A98G-K103N-V108I	1 (2.4)	HLR	HLR	S^#^	S
V106A-P225H	1 (2.4)	HLR	HLR	S	S
K103N only (or with P225H or V106A)	4 (9.8)	HLR	HLR	S	S
Y181C only (or with V108I)	4 (9.8)	IR	HLR	IR	IR
G190A-K103N-K101E/Q	2 (4.9)	IR	HLR	LLR	LLR
G190A	1 (2.4)	IR	HLR	PLR	PLR
V106A	1 (2.4)	IR	HLR	S	S
V179E	2 (4.9)	PLR	PLR	PLR	PLR
V108I	1 (2.4)	PLR	LLR	S	S
K101Q	1 (2.4)	S	S	S	S
**ART-Naïve (n = 17)**					
G190A-Y181C	1 (2.4)	HLR	HLR	HLR	HLR
G190A-K101E	1 (2.4)	HLR	HLR	LLR	LLR
K103N	2 (4.9)	HLR	HLR	S	S
Y181C	2 (4.9)	IR	HLR	IR	IR
P225H	1 (2.4)	IR	IR	S	S
V179E	4 (9.8)	PLR	PLR	PLR	PLR
V108I	1 (2.4)	PLR	LLR	S	S
V106, V118I	1 (2.4)	S	PLR	PLR	PLR
E138A	2 (4.9)	S	S	PLR	LLR
V90I	1 (2.4)	S	S	S	S
K101Q	1 (2.4)	S	S	S	S

^&^EFV: Efavirenz

^š^ETR: Etravirine

^¶^ NVP: Nevirapine

^μ^RPV: Rilpivirine

^#^S: susceptible

* HLR: High-level resistance

^†^IR: Intermediate resistance

^ø^LLR: Low-level resistance

^‡^PLR: Potential low-level resistance

### Resistance levels to various antiretroviral drugs among ART-experienced patients

**Fig 3:**
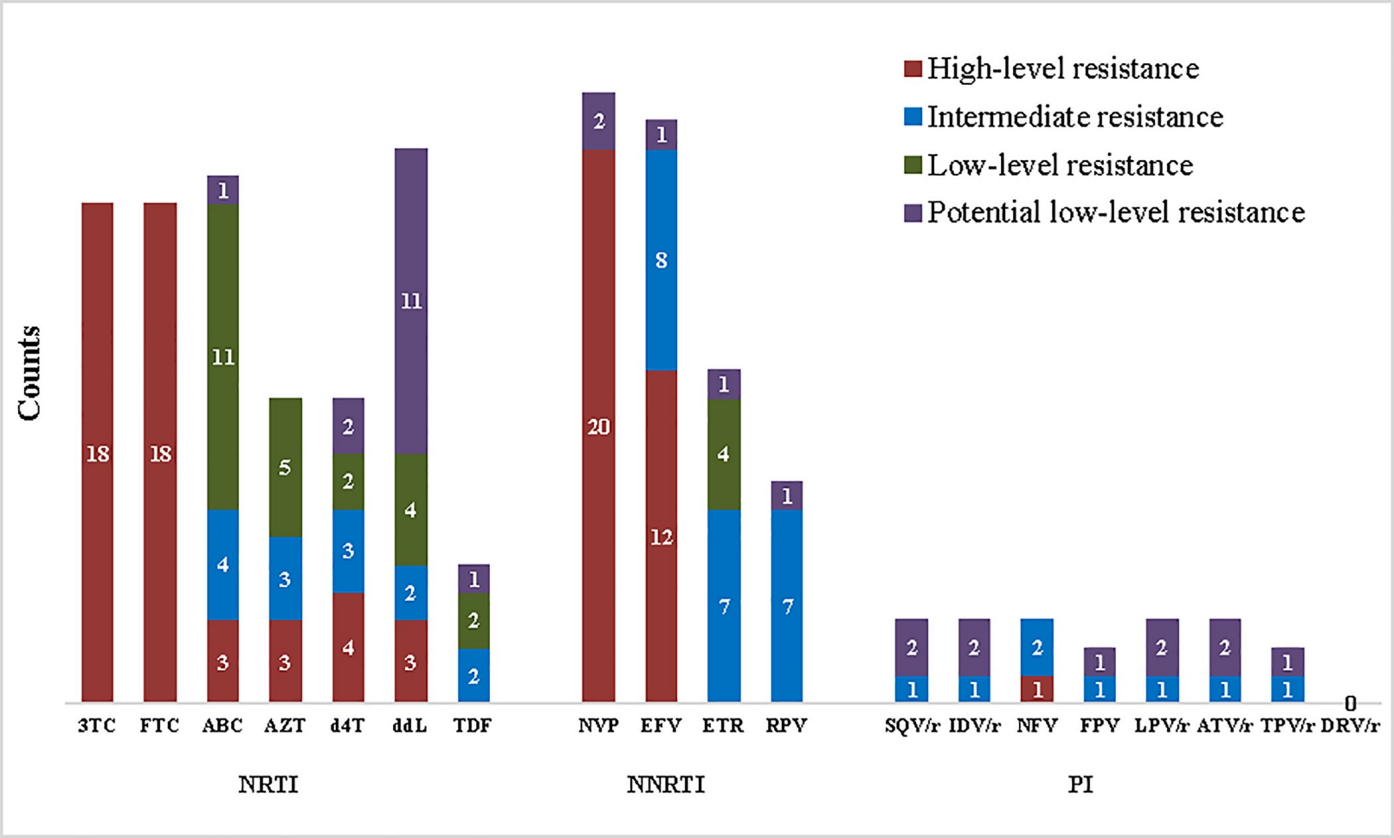
Frequency and levels of HIV-1 drug resistance to various antiretroviral drugs among ART-experienced patients. NRTI: Nucleotide reverse transcriptase inhibitors [3TC: lamivudine; FTC: emtricitabine; ABC: abacavir; AZT: zidovudine; d4T: stavudine; ddl: didanosine; TDF: tenofovir]. NNRTI: Non-Nucleoside reverse transcriptase inhibitors [NVP: nevirapine; EFV: efavirenz; ETR: etravirine; RPV: rilpivirine]. PI: Protease inhibitors [SQV/r: saquinavir; IDV/r: indinavir FPV/r: fosamprenavir; LPV/r: lopinavir; ATV/r: atazanavir]. Red represent high-level resistance, blue represent intermediate resistance, green represent low-level resistance and purple represent potential low-level resistance.

Of the 21 sequences with NRTI-associated mutations, 18 (85.7%) exhibited high-level resistance (HLR) to both lamivudine and emtricitabine. Tenofovir was the least resistant drug with 2 (9.5%) sequences each exhibiting intermediate and low-level resistance and 1 (4.8%) potential low-level resistance (Fig 3). On the other hand, of the 24 sequences with NNRTI-associated mutations, 20 (83.3%) and 12 (50%) demonstrated HLR to nevirapine and efavirenz respectively. Etravirine and rilpivirine were the least resistant (Fig 3).

The three sequences with major protease inhibitor (PI) mutations were from ART-experienced patients. The sequence with the non-polymorphic PI-selected mutations (I54T and V82A) confers HLR to nelfinavir and intermediate resistance to all other PI drugs except darunavir. Mutations M46I and M46LV demonstrated potential low-level resistance to saquinavir, indinavir, fosamprenavir, lopinavir and atazanavir (Fig 3).

### Resistance levels to various antiretroviral drugs among ART-naïve patients

**Fig 4:**
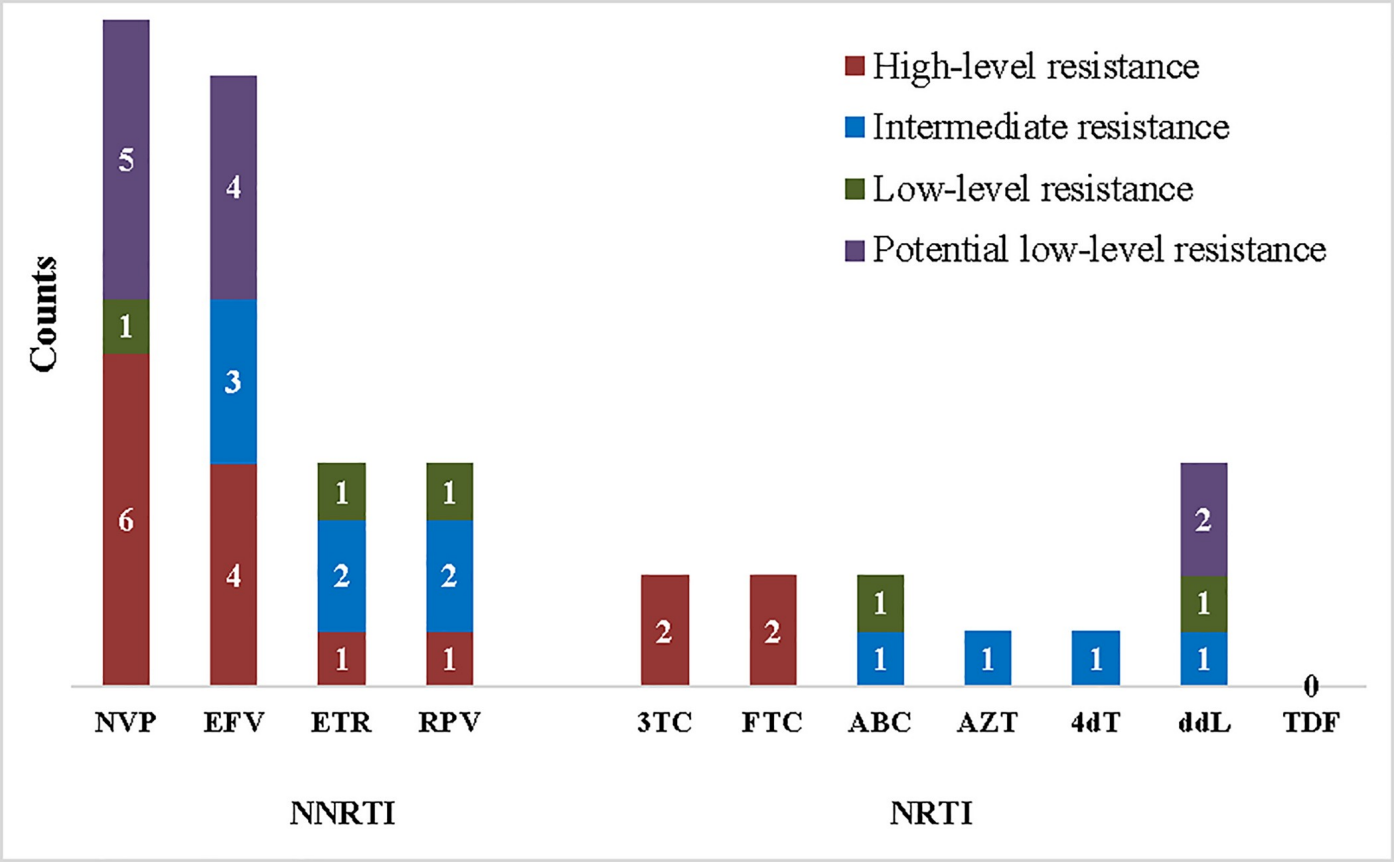
Frequency and levels of HIV-1 drug resistance to various antiretroviral drugs among ART-naïve patients. NRTI: Nucleotide reverse transcriptase inhibitors [3TC: lamivudine; FTC: emtricitabine; ABC: abacavir; AZT: zidovudine; d4T: stavudine; ddl: didanosine; TDF: tenofovir]. NNRTI: Non-Nucleoside reverse transcriptase inhibitors [NVP: nevirapine; EFV: efavirenz; ETR: etravirine; RPV: rilpivirine]. Red represent high-level resistance, blue represent intermediate resistance, green represent low-level resistance and purple represent potential low-level resistance.

Among ART-naïve patients, only 4 sequences had NRTI-associated mutations, two had HLR to lamivudine and two to emtricitabine. While of the 17 sequences with NNRTI-associated mutations, 6 (35.3%) and 4 (23.5%) of them showed high-level resistance to nevirapine and efavirenz respectively (Fig 4).

### Factors associated with the occurrence of drug resistance-associated mutations in ART-experienced patients

In ART-experienced participants, the occurrence of drug resistance mutation was not influenced by most socioeconomic and demographic variables. However, participants ≤ 7 years of formal education were more than 3-times at risk of having resistant mutations than those with more than 7 years of formal education (56.0% versus 26.7%, p = 0.015). Similarly, resistant mutations were common among participants with a low monthly income of ≤ 50,000 XAF (≤100 USD), although not significant (42.1% versus 15.4%, p = 0.072) (Table 4).

**Table 4:** Factors associated with the occurrence of drug resistance mutations in ART-experienced patients.

Factors Categories		Prevalence of mutation, n (%)			
**Demographic**		**Presence**	**Absence**	^μ^**OR (^¶^CI),** **p-value**	^&^**aOR (CI),** **p-value**
Gender	Male	4 (23.5)	13 (76.5)	0.43 (0.13–1.51), 0.182	
	Female	22 (41.5)	31 (58.5)	1	
Age (years)	19–29	5 (25.0)	15 (75.0)	0.33 (0.08–1.36), 0.126	
	30–40	13 (38.2)	21 (61.8)	0.65 (0.20–2.05), 0.433	
	> 40	8 (50.0)	8 (50.0)	1	
Region	Northwest	8 (47.1)	9 (52.9)	1.72 (0.57–5.24), 0.331	
	Southwest	18 (34.0)	35 (66.0)	1	
A treatment centre in the municipality	Out	19 (41.3)	27 (58.7)	1.71 (0.59–4.92), 0.318	
	Within	7 (29.2)	17 (70.8)	1	
**Socio-economic**		** **	** **	** **	
Level of education (years of school)	Low (≤ 7)	14 (56.0)	11 (44.0)	**3.5 (1.25–9.8), 0.015**	2.77 (0.88–8.76), 0.082
	High (> 7)	12 (26.7)	33 (73.3)	1	1
Income level (XAF) (50000 ~100 USD)	≤ 50,000	24 (42.1)	33 (57.9)	4.0 (0.81–19.73) 0.072	
	> 50,000	2 (15.4)	11 (84.6)	1	
Alcohol use	Yes	5 (21.7)	18 (78.3)	0.34 (0.11–1.08), 0.062	
	No	21 (44.7)	26 (55.3)	1	
Smoking	Yes	3 (50.0))	3 (50.0)	1.78 (0.33–9.56), 0.495	
	No	23 (35.9)	41 (64.1)	1	
**Clinical history**		** **	** **	** **	
Duration of ART	≥ 12 months	19 (54.3)	16 (45.7)	**4.75 (1.64–13.74), 0.003**	**4.07 (1.05–15.72), 0.042**
	< 12 months	7 (20.0)	28 (80.0)	1	1
Regimen base	NVP-based	18 (40.9)	26 (59.1)	1.56 (0.56–4.35), 0.396	
	EFV- based	8 (30.8)	18 (69.2)	1	
CD4+ status (cell/μl)	< 200	13 (46.4)	15 (53.6)	2.60 (0.58–11.69), 0.213	
	200–499	10 (37.0)	17 (63.0)	1.77 (0.39–8.09), 0.465)	
	≥ 500	3 (25.0)	9 (75.0)	1	
HIV Log_10_ viral load	≥ 3.6 copies/ml	15 (50.0)	15 (50.0)	2.64 (0.97–7.14), 0.054	
	< 3.6 copies/ml	11 (27.5)	29 (72.5)	1	
HIV subtypes	Single forms	11 (61.1)	7 (38.9)	**3.88 (1.26–11.90), 0.015**	1.68 (0.43–6.56), 0.456
	Recombinant	15 (28.8)	37 (71.2)	1	

^¶^CI: confidence interval

^μ^OR: Odds ratio

^&^aOR: adjusted odds ratio

In terms of clinical factors, the occurrence of resistant mutations increased with the duration of antiretroviral therapy (ART). Patients on ART for ≥ 12 months were more likely to develop resistant mutations compared to those of < 12 months (OR: 5.32; 95% CI: 1.62–17.43, p = 0.006). Though not significant, the occurrence of resistant mutations were common among patients with CD4 count < 200 cells/μl (OR: 2.60 CI:0.58–11.69, p = 0.213) and those with viraemic levels of ≥ 3.6 log_10_ copies/ml (OR: 2.64 CI: 0.97–7.14, p = 0.054). Resistance was also significantly prominent among patients carrying non-recombinant HIV-1 subtypes than recombinant forms (OR: 3.88, CI: 1.26–11.90, p = 0.015).

In a multivariable logistic regression analysis that included all significant univariate variables, duration on ART (OR: 4.07, CI: 1.05–15.72, p = 0.042) was the lone variable that was independently associated with the occurrence of resistant mutations. The level of education was borderline (OR: 2.77, CI: 0.88–8.76, p = 0.082) independent predictor of the occurrence of resistant mutations (Table 4).

### Factors associated with the occurrence of drug resistance-associated mutations in ART-naive patients

Among ART-naïve patients, all the variables analysed except of viral load, did not significantly impact the occurrence of drug resistance-associated mutations. Participants with a log_10_ viral load level of ≥ 5.2 copies/ml had higher chances (p = 0.007) of having drug resistant-mutations. On the other hand, pre-treatment resistant mutations were higher among male than female (13.8% versus 8.7%, p = 0.287), among participants from Northwest than Southwest region (15.1% versus 8.6% p = 0.206), those whose treatment centres were out of the municipality than within (14.3% versus 6.4%, p = 0.098) and among those with low level of education (14.3% versus 7.1%, p = 0.128). However, none of these variables was significant and the numbers of participants were small, hence increasing the possibility of type II statistical error (Table 5).

**Table 5:** Factors associated with the occurrence of drug resistance-associated mutations in ART-naive patients.

Factors Category		Prevalence of mutation, n (%)		
**Demographic**		**Presence**	**Absence**	**OR (CI), p-value**
Gender	Male	9 (13.8)	56 (86.2)	1.70 (0.64–4.53), 0.287
	Female	9 (8.7)	95 (91.3)	1
Age (years)	19–29	5 (9.1)	50 (90.9)	0.51 (0.15–1.75), 0.287
	30–40	6 (8.5)	65 (91.5)	0.48 (0.15–1.52), 0.210
	> 40	7 (16.3)	36 (83.7)	1
Region	Northwest	8 (15.1)	45 (84.9)	1.88 (0.70–5.09), 0.206
	Southwest	10 (8.6)	106 (91.4)	1
A treatment centre in the municipality	Out	13 (14.3)	78 (85.7)	2.43 (0.83–7.16), 0.098
	Within	5 (6.4)	73 (93.6	1
**Socio-economic**		** **	** **	** **
Level of education	Low (≤ 7)	12 (14.3)	72 (85.7)	2.19 (0.78–6.15), 0.128
(years of school)	High (> 7)	6 (7.1)	79 (92.9)	
Income level (XAF)	≤ 50,000	12 (9.2)	119 (90.8)	0.54 (0.19–1.54), 0.243
(50000 ~100 USD)	> 50,000	6 (15.8)	32 (84.2)	1
**Behavioural**		** **	** **	** **
Alcohol use	Yes	9 (11.5)	69 (88.5)	1.19 (0.45–3.16), 0.729
	No	9 (9.9)	82 (90.1)	1
Smoking	Yes	2 (16.7)	10 (83.3)	1.76 (0.35–8.76), 0.483
	No	16 (10.2)	141 (89.8)	1
**Clinical history**		** **		
CD4+ status (cells/ml)	< 200	2 (5.9)	32 (94.1)	0.97 (0.13–7.31), 0.975
	200–499	5 (10.4)	43 (89.6)	1.80 (0.33–9.90), 0.498
	≥ 500	2 (6.1)	31 (93.9)	1
HIV subtypes	Single forms	4 (10.3)	35 (89.7)	0.95 (0.29–3.06), 0.927
	Recombinant	14 (10.8)	116 (89.2)	1
HIV Log_10_ viral load	≥ 5.2 copies/ml	15 (17.6)	70 (82.4)	**5.79 (1.61–20.8), 0.007**
	< 5.2 copies/ml	3 (3.6)	81 (96.4)	1

## Discussion

HIV-1 genetic diversity is paramount in assessing treatment strategies, response to treatment and surveillance of drug resistance. In tracking the evolution of the HIV-1 pandemic, differences in subtypes continue to play an important role [21],while HIV drug resistance surveillance is vital to build and sustain the benefits in ART scale-up, towards achieving UNAIDS 90-90-90 targets [30]. In our study area, subtype CRF02 (A/G) continues to predominate, followed by G and F as seen in other studies conducted in different parts of Cameroon [17,31–34].

As previously described [35–37], M184V was the most prevalent NRTI mutation occurring in all the 10 major mutational patterns reported in this study. M184V is associated with high-level resistance to lamivudine and emtricitabine. K103N also was the most prevalent NNRTI mutation and is responsible for cross-resistance in this drug class. The second prevalent resistance-associated mutations were T215Y/F and Y181C in the NRTI and NNRTI respectively. These mutations correspond to those reported in the literature [38,39] and are extremely important as they result in cross-resistance within the same class of antiretrovirals.

The low prevalence (1.3%) of major protease inhibitor mutations reflect the low usage of these drugs at the time of the study. Moreover, even in treatment naïve patients, natural polymorphisms during HIV replication could give rise to some viruses already harbouring mutations that confer resistance to protease inhibitors [40,41]. These HIV polymorphisms are inevitable due to the high viral replication rate [42] and the poor proofreading ability of reverse transcriptase [42], which virtually generates an assorted pool of viral variants [40]. These groups of mutant viruses although small in number could gradually become the dominant population if the patient is put on an ART regimen that includes a protease inhibitor.

The highest resistance (according to HIV db program) was seen with NNRTI drugs such as nevirapine, with a low genetic barrier [43]. Nevirapine was used as monotherapy in the prevention of mother-child transmission in Cameroon. Frequent emergence of nevirapine-associated mutations has been reported in women exposed to single-dose nevirapine [44]. Etravirine and rilpivirine demonstrated the least resistance among the NNRTIs. Although these drugs were not among the approved regimens used in Cameroon at the time of this study, they already showed some level of resistance as both had 15.7% intermediate resistance in ART-experienced patients. Tenofovir had the least resistance among patients on ART [45] and no resistance was reported among treatment-naïve patients probably due to fact that this drug was recently introduced in Cameroon in 2010, as a replacement to stavudine [46]. Resistance to lamivudine and emtricitabine were high in this study, yet lamivudine remains critical to recent innovative treatment strategies due to its efficacy, safety profile and the availability of low-cost generic versions [47].

Socioeconomic and demographic predictors of resistance are linked to adherence issues [48][49] that are necessary to maintain an undetectable viral load level. Non-adherence results in “drug holiday” which has been shown to predict treatment failure [50] and is associated with virologic rebound and emergence of drug-resistant viruses. A review of available data does not provide conclusive support for the existence of a clear-cut association between socioeconomic status (SES) and adherence of HIV/AIDS patients. However, there seems to be a positive trend with some components of SES such as income, education, and occupation [27]. This study was limited because adherence was not assessed. Nevertheless, most socioeconomic variables did not also show any significant association with the occurrence of resistant mutations in both ART-naïve and ART-experienced patients. However, in line with a study conducted in China [47], a lower level of education (≤ 7 years of school) was associated (p = 0.015) with resistance-associated mutations amongst ART-experienced patients. While low monthly income (≤ 50,000 XAF) was borderline associated (p = 0.072) with the occurrence of resistance mutations.

Furthermore, although not significant, participants who had to cover longer distances to the treatment centers were more at risk of having resistance-associated mutations. The social construction and mental representations of HIV as a stigmatising condition have created significant barriers to HIV testing, protective behaviours, access to treatment and adherence and management of the disease [51]. In this light, due to stigmatisation, patients avoid close-by treatment centers/units and choose to seek care at far-off treatment centers. Additionally, because a larger proportion of our study participants fell in the low-income class (≤ 50,000 XAF or ≤ 100 US dollars/month) [52], they could have missed their clinic and drug refill rendezvous due to financial difficulties and distance barrier, thereby resulting in "drug holidays". A previous study carried out in Kenya had reported the association of pre-treatment drug resistance with unemployment among ART-experienced patients [53].

Clinical predictors such as ART duration (p = 0.006) and HIV subtypes (p = 0.015) significantly predicted the occurrence of resistance mutations in ART-experienced patients. Exposure to ART is an important risk factor for developing resistance. The incidence increases with prolonged exposure [54] and therefore, those who had been on ART for more than 12 months were more at risk in the current study. However, duration on ART was the lone independently predictor of the occurrence of resistant mutations as per the multivariable logistic regression analysis. There are contradictory concering the association between drug resistance and HIV subtypes [55]. The occurrence of resistant mutations was common among patients infected with non-recombinant HIV subtypes as compared to those with recombinant forms in our study. A previous study [56] showed a significant association between subtype C and the emergence of resistant mutations. Santos *et al*. demonstrated the susceptibility of CRF02 (A/G) subtype to protease inhibitors such as nelfinavir and ritonavir when compared to B, C, F and G [57]. Unfortunately, of all the different subtypes in existence, only subtype B has been extensively studied in terms of drug resistance [56,58]. In line with other studies [55,59], the occurrence of resistant mutations were common among patients with CD4 count < 200 cells/μl and those with viraemic levels of ≥ 3.6 log_10_ copies/ml in ART-experienced patients, although it wasn’t significant, while only viral load was associated with the occurrence of resistance mutation among ART-naive patients in a univariate analysis.

We have to acknowledge there were some limitations to this study. Firstly, it was a cross-sectional study, which included all participants present at the time of enrolment irrespective of the duration of treatment. Therefore, the study was not conducted following the WHO current guidelines for transmitted and pretreatment drug resistance surveys. Secondly, we could not ascertain prior ART exposure among participants, including through the prevention of mother-to-child transmission (PMTCT) programme in female participants. Thirdly, adherence to ART was not also assessed. Finally, given the short duration of ART and the small sample size of ART-exposed participants, the predictors of acquired drug resistance cannot be generalized.

## Conclusion

In conclusion, 69% of HIV subtypes circulating in these regions are CRF_02 (A/G). The high resistance to NNRTI, which are the main support to the backbone (NRTI) first-line antiretroviral regimens in Cameroon, has prompted the need to rollout an integrase strand transfer inhibitor regimen (containing Dolutegravir) with a higher genetic barrier to resistance as the preferred first-line regimen.

## Supporting information

S1 File Dataset Genetic diversity and mutations profiles. (XLSX)
